# Identification of Amino Acid Residues of ERH Required for Its Recruitment to Nuclear Speckles and Replication Foci in HeLa Cells

**DOI:** 10.1371/journal.pone.0074885

**Published:** 2013-08-28

**Authors:** Monika I. Banko, Marek K. Krzyzanowski, Paulina Turcza, Zuzanna Maniecka, Marta Kulis, Piotr Kozlowski

**Affiliations:** Department of Molecular Biology, Faculty of Biology, University of Warsaw, Warsaw, Poland; Russian Academy of Sciences, Institute for Biological Instrumentation, Russian Federation

## Abstract

ERH is a small, highly evolutionarily conserved nuclear protein of unknown function. Its three-dimensional structure is absolutely unique and it can form a homodimer through a β sheet surface. ERH has been shown to interact, among others, with PDIP46/SKAR and Ciz1. When coexpressed with the latter protein, ERH accumulates in replication foci in the nucleus of HeLa cells. Here, we report that when ERH is coexpressed with PDIP46/SKAR in HeLa cells, it is recruited to nuclear speckles, and identify amino acid residues critical for targeting ERH to both these subnuclear structures. ERH H3A Q9A shows a diminished recruitment to nuclear speckles but it is recruited to replication foci. ERH E37A T51A is very poorly recruited to replication foci while still accumulating in nuclear speckles. Consequently, ERH H3A Q9A E37A T51A is recruited neither to nuclear speckles nor to replication foci. The lack of interactions of these three ERH forms with PDIP46/SKAR and/or Ciz1 was further confirmed *in vitro* by GST pull-down assay. The residues whose substitutions interfere with the accumulation in nuclear speckles are situated on the β sheet surface of ERH, indicating that only the monomer of ERH can interact with PDIP46/SKAR. Substitutions affecting the recruitment to replication foci map to the other side of ERH, near a long loop between the α1 and α2 helices, thus both the monomer and the dimer of ERH could interact with Ciz1. The construction of the ERH mutants not recruited to nuclear speckles or replication foci will facilitate further studies on ERH actions in these subnuclear structures.

## Introduction

The enhancer of rudimentary homolog (ERH) gene encoding a small protein of approx. 100 amino acid residues is widely present in animals, plants, and protists, but only in some fungi (generally, not in the higher fungi, with the prominent exception of *Schizosaccharomyces pombe*) [1–6]. The amino acid sequence of ERH is highly conserved evolutionarily but it is not similar to any other protein and is completely uninformative regarding its putative function or intracellular localization [2,6]. The three-dimensional structure of ERH has been studied extensively [6–9]. The polypeptide chain forms a unique α + β fold with three α helices situated on one side of a four-stranded β sheet with the topology β1-3_10_-β2-α1-α2-β3-β4-α3 [6–9]. In addition, ERH can function as a homodimer in which two monomers stick to each other through the β sheet surfaces [6–9]. ERH localizes in a diffused pattern to the nucleus with the exception of the nucleoli [10]. *ERH* is expressed at a roughly constant level in all normal adult and fetal human tissues, however, it is up regulated in human breast, ovarian, and liver cancers [10,11].

The exact function of ERH is unknown but some have been proposed based on its genetic or biochemical interactions. Firstly, the *ERH* gene was identified in *Drosophila melanogaster* as a mutation augmenting the truncated wing phenotype of hypomorphic mutations in the *rudimentary* gene which codes for a protein with the first three enzymatic activities of the *de novo* pyrimidine nucleotide biosynthetic pathway [1,12]. Hence, the novel gene was named *enhancer of rudimentary* and its protein product was proposed to play some role in the regulation of pyrimidine metabolism [1]. Later, the same group suggested that ERH could be involved in the regulation of the cell cycle and cellular proliferation, and also in the regulation of the Notch signaling pathway [2,13]. In *Xenopus laevis*, ERH interacts with DCoH/PCD, a dimerization cofactor of the HNF1 transcription factor, acting as a transcriptional repressor, but this phenomenon seems to be tissue-specific since DCoH/PCD is detected mainly in the liver and kidneys [4]. We showed that human ERH interacts with two nuclear proteins, Ciz1 and PDIP46/SKAR [10,14]. The genes coding for these molecular partners are expressed in all major adult and fetal tissues, thus these two interactions can take place ubiquitously in the human organism throughout its whole life [10,14]. Initially, Ciz1 was identified as an interactor of p21^Cip1/Waf1^, a protein inhibitor of cyclin-dependent kinases [15]. Later, it was found to function as a DNA replication factor [16]. Ciz1 is present in replication foci, and when coexpressed with ERH in HeLa cells, it recruits ERH to these sites [14,16]. The other molecular partner, PDIP46/SKAR, was originally identified as an interactor of the p50 subunit of Pol δ, the lagging strand DNA polymerase [17]. It was also identified as an interactor of S6K1, a protein kinase regulating cell growth [18]. Subsequently, it was found to localize to nuclear speckles, structures that store splicing factors, and to be a component of the exon junction complex recruiting activated S6K1 which in turn increases the translational efficiency of newly spliced mRNAs by phosphorylation of mRNP-associated proteins [18,19]. The regions of Ciz1 and PDIP46/SKAR required for the interactions with ERH have been determined and they overlap with the regions involved in their interactions with p21^Cip1/Waf1^ and S6K1, respectively [10,14,15,18]. Unfortunately, the three-dimensional structures of Ciz1 and PDIP46/SKAR have not been resolved yet. Studies on other proteins and a high-throughput analysis have indicated nuclear proteins SPT5, FCP1, MED31, TLE1, and HOTS, among others, as interactors of ERH [20–23]. Although functions of some of them are known, notably the first four proteins have a role in transcription, the very interactions with ERH have not been subjected to more detailed studies yet. Recently, it was reported that, through interaction with the spliceosomal protein SNRPD3, ERH controls pre-mRNA splicing of CENP-E, a kinesin required for the proper kinetochore-microtubule attachments and alignment of chromosomes at the metaphase plate during mitosis [24,25]. The biological activity of ERH seems to be regulated by CK2 kinase, which can phosphorylate threonine 18 and serine 24 in *Drosophila* ERH *in vitro* [26]. These residues are conserved in most ERHs and solvent exposed, and their phosphorylation is predicted to disrupt ERH dimerization and interactions with other proteins [6].

Here, we refined the subcellular localization of human ERH and its interaction with PDIP46/SKAR in HeLa cells, and identified the ERH amino acid residues required for its recruitment to nuclear speckles and replication foci in these cells by PDIP46/SKAR and Ciz1, respectively.

## Results

### Subcellular localization of ERH

**Figure 1:**
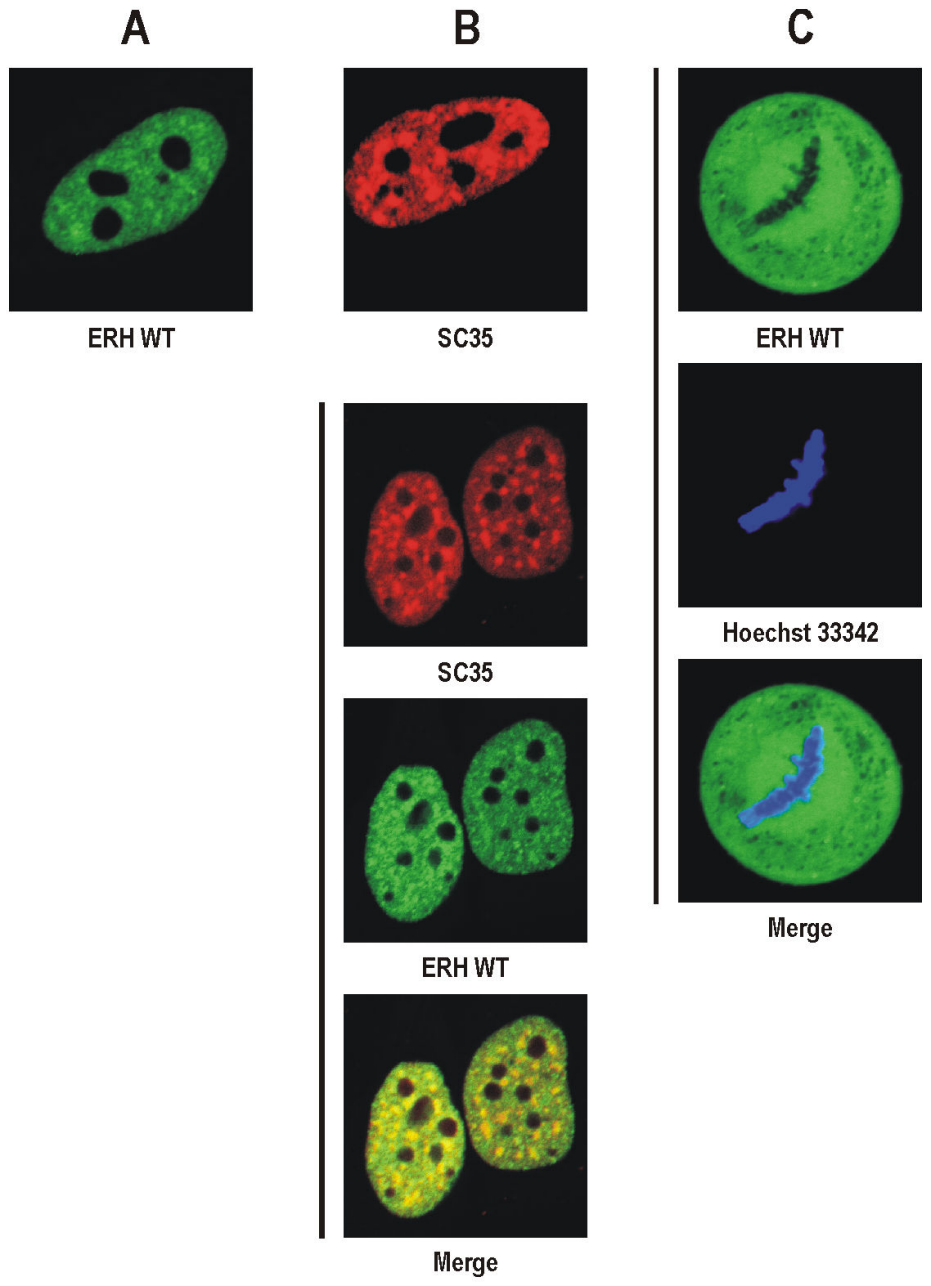
Subcellular localization of human ERH in HeLa cells visualized by confocal microscopy. **A**. EGFP-tagged wild-type ERH in the nucleus. ERH is present in the nucleoplasm where it can form weak spots. **B**. mCherry-tagged human SC35 expressed alone (top) or coexpressed with EGFP-tagged wild-type ERH (bottom). SC35 accumulates in nuclear speckles. ERH spots coincide with nuclear speckles. **C**. EGFP-tagged wild-type ERH in the metaphase cell with chromosomes stained with Hoechst 33342. ERH is distributed uniformly throughout the cytoplasm excluding space occupied by the condensed chromosomes.

We reported previously that human ERH localizes in a diffused pattern to the nucleus of HeLa cells excluding the nucleoli [10]. However, at closer examination we found that in some nuclei ERH also forms weak spots in the nucleoplasm (Figure 1A). The number, shape and distribution of these spots resembled those of nuclear speckles, for which the SC35 protein is a molecular marker (Figure 1B) [27]. To establish the nature of the ERH spots in the nucleus we tested colocalization of EGFP-tagged human ERH and mCherry-tagged human SC35 in HeLa cells. As shown in Figure 1B the ERH dots coincided with the SC35 dots. However, ERH did not seem to be present in nuclear speckles due to a direct interaction with SC35 since these proteins do not interact with each other when tested in the yeast two-hybrid (Y2-H) system (data not shown).

We also investigated the subcellular localization of EGFP-tagged human ERH throughout the cell cycle in HeLa cells and found that during mitosis ERH is present in the cytoplasm, being fully excluded from the space occupied by the condensed chromosomes (Figure 1C).

### Analysis of ERH-PDIP46/SKAR interaction

**Figure 2:**
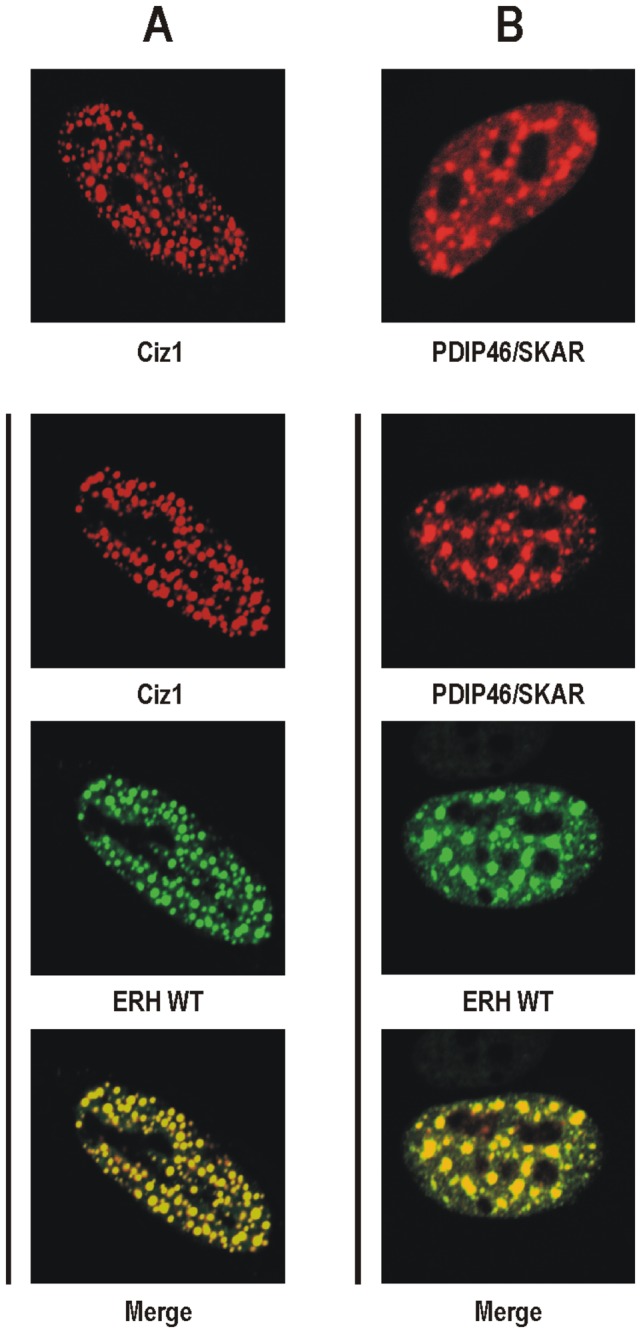
Recruitment of human ERH to nuclear speckles in HeLa cells visualized by confocal microscopy. **A**. mCherry-tagged human Ciz1 expressed alone (top) or coexpressed with EGFP-tagged wild-type ERH (bottom). Ciz1 is present in replication foci and ERH is recruited to these subnuclear structures when coexpressed with Ciz1. **B**. mCherry-tagged human PDIP46/SKAR expressed alone (top) or coexpressed with EGFP-tagged wild-type ERH (bottom). PDIP46/SKAR accumulates in nuclear speckles and ERH is recruited to these subnuclear structures when coexpressed with PDIP46/SKAR.

We demonstrated previously, using the Y2-H system and *in vitro* methods that human ERH interacts with the nuclear protein PDIP46/SKAR [10]. Others showed that PDIP46/SKAR localizes to nuclear speckles [18]. We also showed that when human ERH and Ciz1, another nuclear interactor of ERH, are coexpressed in HeLa cells, ERH is recruited to the Ciz1-containing replication foci (Figure 2A) [14]. To verify the interaction between ERH and PDIP46/SKAR in the living cell we tested whether mCherry-tagged human PDIP46/SKAR can recruit EGFP-tagged human ERH to nuclear speckles in HeLa cells. ERH formed very strong spots in the nucleus of the cells coexpressing PDIP46/SKAR (Figure 2B), which coincided fully with nuclear speckles and were much more intense than those in the nucleus of the cells coexpressing SC35 (Figure 1B).

### Identification of amino acid residues critical for recruitment of ERH to replication foci and nuclear speckles

Identification of the amino acid residues involved in the interactions with the molecular partners is instrumental for better understanding of the functioning of ERH in the cell. Analysis of the human ERH amino acid sequence did not reveal any remarkably interesting residues, apart from T18 and S24, which were shown to be phosphorylated in *Drosophila* ERH by CK2 *in vitro*, and S47 lying within a canonical PxS/TP motif for phosphorylation by MAP kinase [2,28]. However, it is reasonable to expect that residues involved in an interaction with other protein(s) should be present on the surface of a protein molecule rather than buried. Conveniently, the three-dimensional structure of ERH has been resolved experimentally at high resolution, so exact spatial positions of almost all of its residues are known [6–9]. Thus, we decided to examine 26 residues situated on the surface of ERH, including T18, S24 and S47. For the screening, we applied alanine scanning, where an amino acid residue in question is substituted by alanine. Additionally, the potentially phosphorylated residues were changed into aspartic acid to mimic their phosphorylation. Some mutations were combined and, in total, we produced a series of 34 single-, double- or quadruple-substituted forms of ERH by site-directed mutagenesis. All the amino acid residues of ERH analyzed in this study, along with their substitutions, are listed in Table 1. As the interactions of human ERH with Ciz1 and PDIP46/SKAR are the only ones we had been able to verify experimentally, we analyzed interactions between EGFP-tagged ERH and mCherry-tagged PDIP46/SKAR or mCherry-tagged Ciz1 overexpressed in HeLa cells by examining the recruitment of ERH to nuclear speckles or replication foci, respectively.

**Table 1:** Recruitment of substituted forms of human ERH to nuclear speckles and replication foci in HeLa cells.

Recruited to nuclear speckles and replication foci	Not recruited to nuclear speckles but recruited to replication foci	Not recruited to replication foci but recruited to nuclear speckles	Not recruited to nuclear speckles or replication foci
K12A, R13A, E15A,	H3A, Q9A, R17A,	E37A, H39A, K41A,	H3A Q9A E37A T51A
T18A, T18D, Y19A,	D66A,	T51A,	
D21A, S24A, S24D,			
R42A, S47A, S47D,	H3A Q9A	E37A T51A	
D59A, D62A, D63A,			
R73A, D75A, P81A,			
K84A, W86A, E89A,			
T18A S24A,			
T18D S24D			

**Figure 3:**
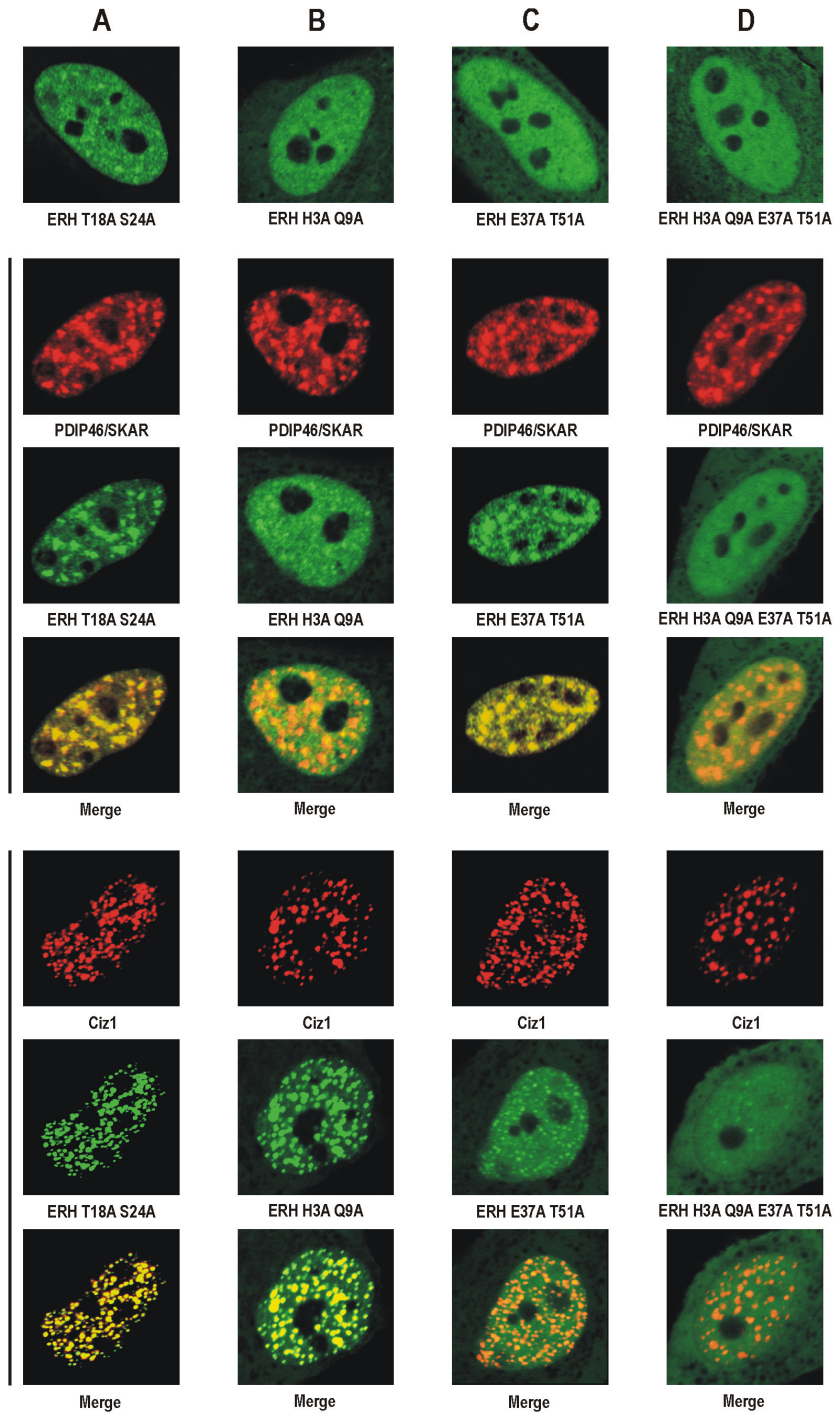
Recruitment of substituted forms of human ERH to nuclear speckles and replication foci in HeLa cells visualized by confocal microscopy. EGFP-tagged substituted forms of ERH expressed alone (top) or coexpressed with mCherry-tagged human PDIP46/SKAR (middle) or mCherry-tagged human Ciz1 (bottom). **A**. ERH T18A S24A localizes to the nucleus and is recruited both to nuclear speckles and to replication foci similarly to wild-type ERH. **B**. ERH H3A Q9A is present not only in the nucleus but also in the cytoplasm, shows diminished recruitment to nuclear speckles but still accumulates in replication foci. **C**. ERH E37A T51A localizes partly to the cytoplasm, is recruited to nuclear speckles, and shows very week accumulation in replication foci. **D**. ERH H3A Q9A E37A T51A is also present in the cytoplasm and recruited neither to nuclear speckles nor to replication foci.

The single-substituted mutant phenotypes could be grouped into three classes (Table 1). Substitution of the majority (18) of the examined residues failed to affect the recruitment of ERH to nuclear speckles or replication foci (data not shown). These included T18, S24 and S47, whether changed to alanine or to aspartic acid. The double-substituted forms T18A S24A (Figure 3A) and T18D S24D (data not shown) also did not show changes in their accumulation in nuclear speckles or replication foci. Substitution of any of four residues (H3, Q9, R17 and D66) resulted in a weaker recruitment of ERH to nuclear speckles, with the strongest effect observed for the H3A and Q9A forms (data not shown). Substitution of any of another four residues (E37, H39, K41 and T51) resulted in a weaker recruitment of ERH to replication foci, with the strongest effect observed for the E37A and T51A forms (data not shown).

To enhance the observed effects we constructed double-substituted forms, H3A Q9A and E37A T51A. H3A Q9A exhibited a minimal ability to accumulate in nuclear speckles whereas it was strongly recruited to replication foci (Figure 3B). E37A T51A exhibited only a very weak ability to accumulate in replication foci but was normally recruited to nuclear speckles (Figure 3C). Finally, we constructed the quadruple-substituted H3A Q9A E37A T51A form, which was negligibly recruited both to nuclear speckles and to replication foci (Figure 3D). Additionally, we observed that some minor fractions of the H3A Q9A, E37A T51A and H3A Q9A E37A T51A forms, but not the T18A S24A form, were also present in the cytoplasm (Figure 3).

**Figure 4:**
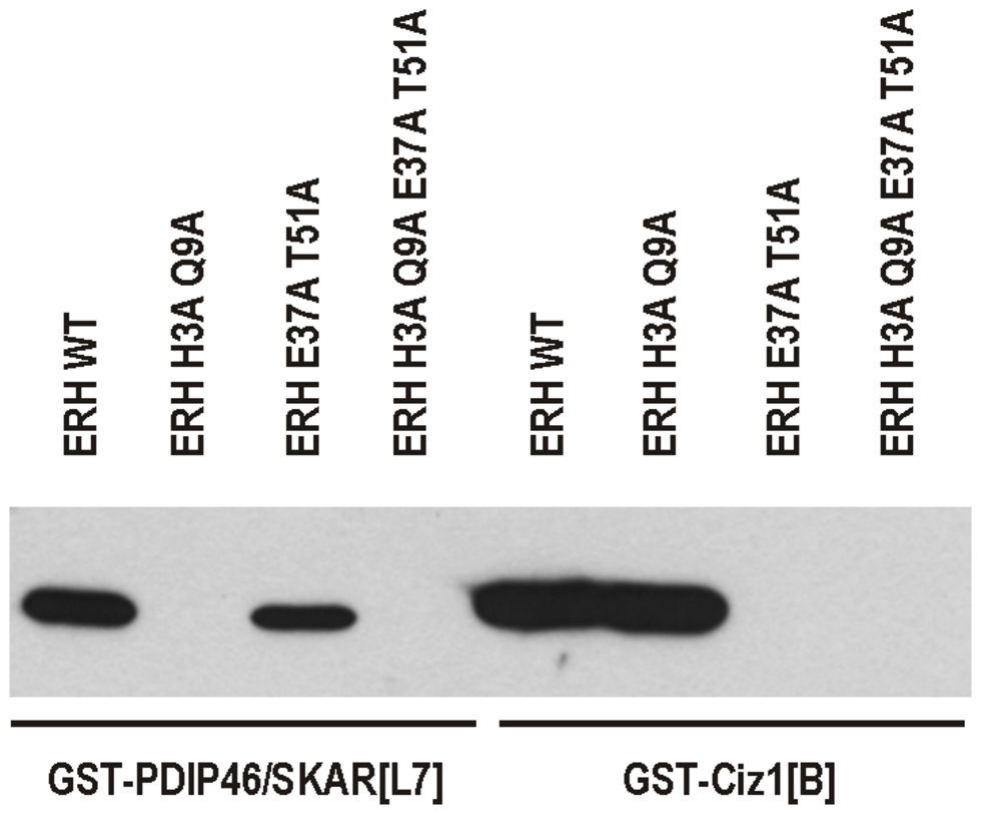
GST pull-down assay with substituted forms of human ERH. Indicated FLAG-tagged ERH forms incubated with either GST-tagged fragment L7 of human PDIP46/SKAR (GST-PDIP46/SKAR[L7]) or GST-tagged fragment B of human Ciz1 (GST-Ciz1[B]) and detected by western blotting with anti-FLAG antibody followed by enhanced chemiluminescence reaction. PDIP46/SKAR does not interact with ERH H3A Q9A or ERH H3A Q9A E37A T51A, and Ciz1 does not interact with ERH E37A T51A or ERH H3A Q9A E37A T51A.

We previously showed that fragment L7 of human PDIP46/SKAR comprising residues 259-421 and fragment B of human Ciz1 comprising residues 531-644, both GST-tagged, were able to pull down FLAG-tagged wild-type human ERH expressed in bacteria [10,14]. To verify that the indicated amino acid residues of ERH are indeed required for the interactions with PDIP46/SKAR or Ciz1, we performed GST pull-down assay with the both double-substituted ERH forms and the quadruple-substituted form, all FLAG-tagged (Figure 4). GST-tagged PDIP46/SKAR[L7] was unable to pull-down H3A Q9A but it was capable of binding to E37A T51A, albeit the interaction was not as strong as that with wild-type ERH. Conversely, GST-tagged Ciz1[B] bound H3A Q9A similarly to wild-type ERH and was unable to pull down E37A T51A. Accordingly, H3A Q9A E37A T51A interacted neither with PDIP46/SKAR[L7] nor with Ciz1[B].

## Discussion

The first report on the subcellular distribution of ERH indicated its cytoplasmic localization [4]. However, it was showed later that ERH predominantly localizes to the nucleus with the exception of the nucleoli [10]. In most nuclei, ERH was present in a diffused pattern but in some it also formed weak spots (Figure 1A). Here, we showed that these spots are nuclear speckles, structures where splicing factors, including SC35, are stored (Figure 1B) [27]. PDIP46/SKAR, an interactor of ERH, also localizes to these subnuclear structures [10,18]. Here, we demonstrated that when ERH and PDIP46/SKAR are coexpressed, ERH is strongly recruited to nuclear speckles (Figure 2B). This finding confirms that both proteins interact *in vivo* and probably the aforementioned weak spots of ERH in the nucleus resulted from the interaction between this protein and the endogenous PDIP46/SKAR.

We also investigated the subcellular localization of ERH throughout the cell cycle. As long as the nuclear compartment was present in the cell, ERH was predominantly nuclear, however, after nuclear envelope breakdown during mitosis, ERH was evenly distributed in the cytoplasm and completely excluded from the condensed chromosomes (Figure 1C). Thus, while ERH seems to be involved in DNA replication and transcription, processes associated with loose chromatin, it is not a component of the condensed mitotic chromosomes.

**Figure 5:**
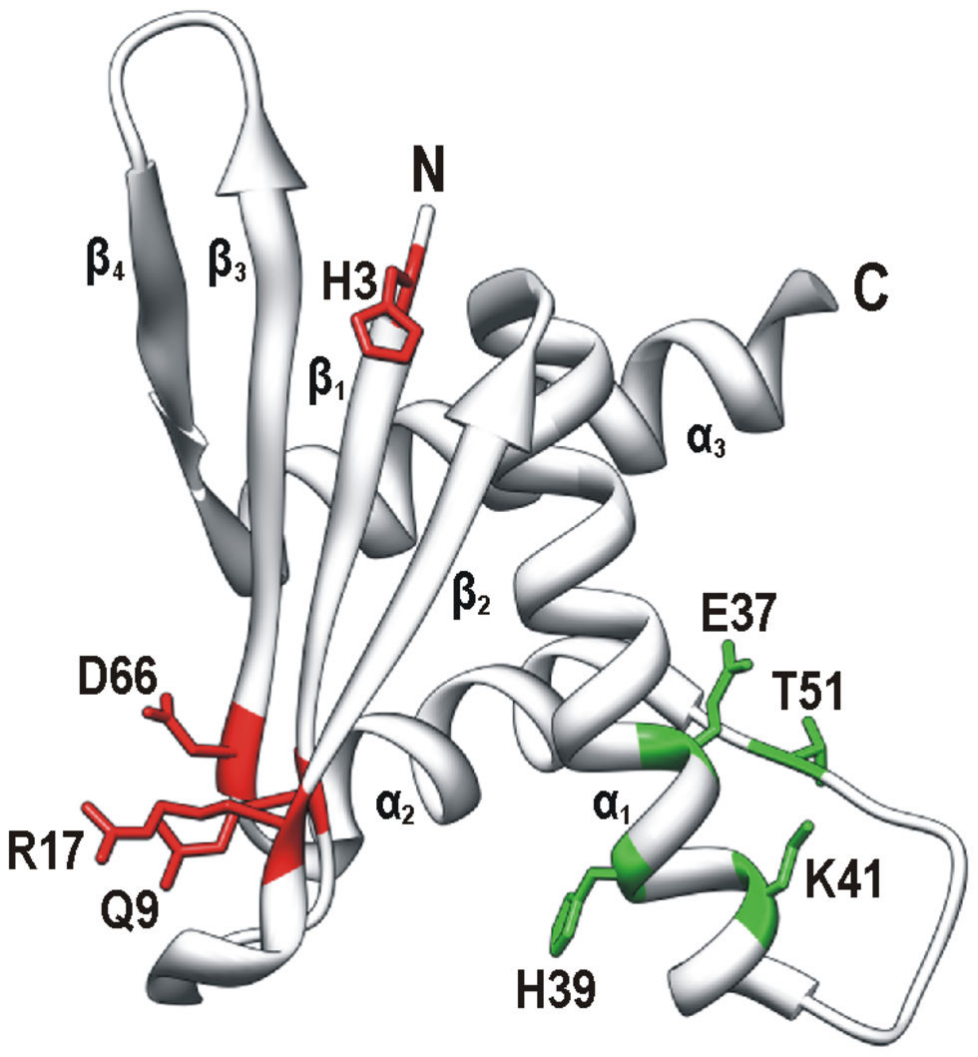
Amino acid residues of human ERH critical for its recruitment to nuclear speckles and replication foci. Three-dimensional structure of a monomer of ERH was produced by UCSF Chimera package using coordinates from Protein Data Bank (2nmlA) [30]. Four β strands (β1, β2, β3 and β4), three α helices (α1, α2 and α3) and the N- and C-termini are indicated. Critical residues are shown with their side chains in color and are labeled using a single-letter code and position number in the polypeptide chain. Residues involved in the recruitment to nuclear speckles (in red) and replication foci (in green) are situated on the β sheet and near the loop between helices α1 and α2, respectively.

Although we do not know the role of ERH in nuclear speckles and replication foci yet, its recruitments to these subnuclear structures can be employed to further characterize this protein *in vivo*. Thus, the primary objective of our work was the identification of the amino acid residues required for the recruitment of ERH to nuclear speckles and replication foci. Employing alanine scanning for residues situated on the surface of ERH we identified two sets of substitutions that interfered with its recruitment to these structures. The substitutions that diminished the recruitment of ERH to nuclear speckles were mapped on the surface of the four-stranded β sheet of ERH (Figure 5). Since ERH accumulates in nuclear speckles via its interaction with PDIP46/SKAR, these substitutions likely disrupted the interaction. However, these substitutions did not interfere with its recruitment to replication foci, indicating that the three-dimensional structure of the protein was not destabilized. In fact, the three-dimensional structure of this single-domain protein appears to be very stable. Substitution of the majority of the examined residues resulted in no change in its behavior in the cell. Moreover, homology modeling showed that even the *S. pombe* homolog Erh1p displaying only 28% identity with human ERH has exactly the same three-dimensional structure [5]. As the GST pull-down assay showed (Figure 4), the double substitution H3A Q9A in ERH abolished completely its binding to PDIP46/SKAR without affecting the binding to Ciz1. Thus, the H3 and Q9 residues of ERH are indeed critical for its interaction with PDIP46/SKAR.

The four-stranded β sheet was suggested to be the ERH homodimer interface [6–9]. Therefore, PDIP46/SKAR could only interact with the ERH monomer. However, since the interaction between ERH and PDIP46/SKAR seems to be rather strong, judging from the Y2-H data, PDIP46/SKAR could simply displace one ERH subunit from the homodimer and bind to the other one. On the other hand, the ERH homodimer was only observed in highly purified preparations of ERH expressed in *Escherichia coli* cells or synthesized by an *E. coli* cell-free protein expression system [6–9], and we have been unable to detect the human ERH homodimer using Y2-H system (M.K.K. and P.K., unpublished data). Thus, the existence of the homodimer *in vivo* remains to be proven.

The substitutions that weakened the recruitment of ERH to replication foci were mapped on the other side of the protein, near the long loop between the α1 and α2 helices (Figure 5). Since ERH seems to be recruited to replication foci through the interaction with Ciz1, these substitutions likely interfered with its binding to this interactor. In contrast, the same substitutions did not seem to hamper the recruitment to nuclear speckles suggesting that the protein could be otherwise functional. The GST pull-down assay (Figure 4) proved that the E37 and T51 residues of ERH are indispensable for its interaction with Ciz1. The interaction with Ciz1 should not be affected by the oligomerization status of ERH, since this surface does not seem to participate in the formation of the homodimer [6–9]. The double-substituted E37A T51A form also exhibited some defect in the binding to PDIP46/SKAR in the GST pull-down assay suggesting that this surface of ERH could be involved in the interaction with PDIP46/SKAR as well. This is plausible because we previously showed that the region of PDIP46/SKAR interacting with ERH has a bipartite nature [10].

Consistently, the quadruple-substituted form in which the residues required for both the recruitment to nuclear speckles and to replication foci were mutated, showed a negligible recruitment to either of these subnuclear structures and was not precipitated in the GST pull-down assay at all.

The primary structure of ERH is highly conserved evolutionarily, especially in Vertebrata in which so distant organisms as humans and *X. laevis* have identical ERH proteins [2,4,6]. Thus, one can expect that almost every amino acid change should impair the functioning of human ERH in the cell. However, most of the substitutions, including those for the potentially phosphorylated residues, did not produce any impact on the protein behavior in our experiments. One explanation of this discrepancy is that these substitutions, neutral in our assay, could turn out to be of importance for some other ERH functions that have not been examined yet, e.g., its interaction with other proteins.

Interestingly, all ERH forms that showed a diminished recruitment to nuclear speckles or to replication foci are also partly defective in the targeting to the nucleus (Figure 3B, C and D). ERH does not possess a nuclear localization signal but, being a small protein with a molecular mass of approx. 12-13 kDa it might cross the nuclear envelope by passive diffusion through the nuclear pores [10]. The appearance of some of the mutated ERH forms in the cytoplasm may indicate that in fact ERH enters the nucleus in a different way, namely, piggyback on its molecular partners possessing a nuclear localization signal. Notably, the quadruple-substituted form that was recruited neither to nuclear speckles nor to replication foci accumulated in the cytoplasm more strongly than did the double mutants (Figure 3D).

ERH seems to function in the cell as a component of several nuclear protein complexes involved in nucleic acid metabolism, which presumably modulates their activities. The identification of the residues critical for its recruitment to the two subnuclear structures is not only an additional step in the verification of the ERH interactions with PDIP46/SKAR and Ciz1 but should also greatly contribute towards better understanding of the mode of action of ERH in the cell, all the more so that owing to the generation of the forms incapable of an interaction with PDIP46/SKAR or with Ciz1, it now becomes possible to study separately the processes in which these proteins participate.

## Materials and Methods

### Plasmids

DNA fragments were amplified by PCR with high fidelity Platinum *Pfx* DNA polymerase (Invitrogene) and cloned using standard methods [29], and generated constructs were verified by automated DNA sequencing. For construction of pmCherry-N1/SC35, an open reading frame of human SC35 was amplified using sense, 5’-GCGAAGCTTCACGATGAGCTACGGCCGCCCCCCT-3’, and antisense, 5’-GCGGGATCCCGAGAGGACACCGCTCCTTCCTC-3’, primers and cloned into the HindIII and BamHI sites of pmCherry-N1 [14]. For construction of pmCherry-N1/PDIP46/SKAR, an open reading frame of the isoform 1/α of human PDIP46/SKAR was amplified using sense, 5’-CGGGGTACCGCAAGATGGCGGACATCTCCCTGGAC-3’, and antisense, 5’-CGGGGTACCGCAAGCTTGATTTTGAATTCTGTG-3’, primers and cloned into the KpnI site of pmCherry-N1. pEGFP-N1/ERH (formerly pEGFP-N1/ER) coding for wild-type human ERH, pmCherry-N1/Ciz1 coding for the longest isoform of human Ciz1, pGEX-4T-1/PDIP46/SKAR[L7] coding for fragment L7 of human PDIP46/SKAR comprising residues 259-421, pGEX-4T-2/Ciz1[B] coding for fragment B of human Ciz1 comprising residues 531-644, and pQE30/ERH-FLAG (formerly pQE30/ER-FLAG) coding for wild-type human ERH were described previously [10,14]. pQE30/ERH[H3A Q9A]-FLAG, pQE30/ERH[E37A T51A]-FLAG, and pQE30/ERH[H3A Q9A E37A T51A]-FLAG were constructed analogously to pQE30/ERH-FLAG using the respective double- and quadruple-substituted pEGFP-N1/ERH constructs (see below) as templates.

### Site-directed mutagenesis of ERH

All reactions were carried out using the QuikChange site-directed mutagenesis method (Stratagene). Briefly, PCR was performed using *PfuTurbo* hotstart DNA polymerase and a pair of mutagenic oligonucleotide primers with a thermal cycling profile: initial denaturation at 95°C for 5 min, 35 cycles of 95°C for 30 s, 52°C for 30 s, and 72°C for 12 min, final extension at 72°C for 10 min, and soak at 4°C. The mutagenic primer pairs are listed in Table 2. The pEGFP-N1/ER plasmid was used as a template for generating single-substituted constructs of ERH. For double- and quadruple-substituted constructs mutations were introduced sequentially. DpnI-treated PCR products were used to transform *Escherichia coli* XL1 Blue MRF' and plasmids were isolated with the NucleoSpin Plasmid kit (Macherey-Nagel). The incorporation of all desired mutations was confirmed by automated DNA sequencing.

**Table 2:** Primers used for generation of substituted forms of human ERH in this study.

Substitution	Sense (5’ → 3’)^a^	Antisense (5’ → 3’)
H3A	AGCTTCACGATGTCTG CCACCATTTTGCTGGTA	TACCAGCAAAATGGTGG CAGACATCGTGAAGCT
Q9A	ACCATTTTGCTGGTAG CGCCTACCAAGAGGCCA	TGGCCTCTTGGTAGGCG CTACCAGCAAAATGGT
K12A	CTGGTACAGCCTACCG CGAGGCCAGAAGGCAG	CTGCCTTCTGGCCTCG CGGTAGGCTGTACCAG
R13A	GTACAGCCTACCAAGG CGCCAGAAGGCAGAAC	GTTCTGCCTTCTGGCG CCTTGGTAGGCTGTAC
E15A	CTACCAAGAGGCCAGCAGGCAGAACTTATGC	GCATAAGTTCTGCCTGCTGGCCTCTTGGTAG
R17A	AAGAGGCCAGAAGGCG CAACTTATGCTGACTAC	GTAGTCAGCATAAGTTG CGCCTTCTGGCCTCTT
T18A	GAGGCCAGAAGGCAGAGCCTATGCTGACTACGAATC	GATTCGTAGTCAGCATAGGCTCTGCCTTCTGGCCTC
T18D	CAAGAGGCCAGAAGGCAGAG ATTATGCTGACTACGAATC	GATTCGTAGTCAGCATAAT CTCTGCCTTCTGGCCTCTTG
Y19A	GCCAGAAGGCAGAACTG CTGCTGACTACGAATCTG	CAGATTCGTAGTCAGCAG CAGTTCTGCCTTCTGGC
D21A	GGCAGAACTTATGCTGCCTACGAATCTGTGAAT	ATTCACAGATTCGTAGGCAGCATAAGTTCTGCC
S24A	CTTATGCTGACTACGAAGCCGTGAATGAATGCATGGAAG	CTTCCATGCATTCATTCACGGCTTCGTAGTCAGCATAAG
T18A S24A^b^	CCTATGCTGACTACGAAGCCGTGAATGAATGCATGGAAG	CTTCCATGCATTCATTCACGGCTTCGTAGTCAGCATAGG
S24D	GAACTTATGCTGACTACGAAG ATGTGAATGAATGCATGGAAGG	CCTTCCATGCATTCATTCACAT CTTCGTAGTCAGCATAAGTTC
T18D S24D^b^	GAG ATTATGCTGACTACGAAG ATGTGAATGAATGCATGGAAGG	CCTTCCATGCATTCATTCACAT CTTCGTAGTCAGCATAAT CTC
E37A	GGTGTTTGTAAAATGTATGCAGAACATCTGAAAAGAATG	CATTCTTTTCAGATGTTCTGCATACATTTTACAAACACC
H39A	GTATGAAGAAG CTCTGAAAAGAATGAATCCCAACAG	CTGTTGGGATTCATTCTTTTCAGAG CTTCTTCATAC
K41A	GTATGAAGAACATCTGG CAAGAATGAATCCCAACAGTCC	GGACTGTTGGGATTCATTCTTG CCAGATGTTCTTCATAC
R42A	GTATGAAGAACATCTGAAAG CAATGAATCCCAACAGTCCC	GGGACTGTTGGGATTCATTG CTTTCAGATGTTCTTCATAC
S47A	CTGAAAAGAATGAATCCCAACG CTCCCTCTATCACATATGACATC	GATGTCATATGTGATAGAGGGAG CGTTGGGATTCATTCTTTTCAG
S47D	CTGAAAAGAATGAATCCCAACG ATCCCTCTATCACATATGACATC	GATGTCATATGTGATAGAGGGATCGTTGGGATTCATTCTTTTCAG
T51A	CCAACAGTCCCTCTATCGCATATGACATCAGTCAG	CTGACTGATGTCATATGCGATAGAGGGACTGTTGG
D59A	ATCAGTCAGTTGTTTGCTTTCATCGATGATCTG	CAGATCATCGATGAAAGCAAACAACTGACTGAT
D62A	TTGTTTGATTTCATCGCTGATCTGGCAGACCTC	GAGGTCTGCCAGATCAGCGATGAAATCAAACAA
D63A	GTTTGATTTCATCGATGCTCTGGCAGACCTCAGCTG	CAGCTGAGGTCTGCCAGAGCATCGATGAAATCAAAC
D66A	ATCGATGATCTGGCAGCCCTCAGCTGCCTGGTT	AACCAGGCAGCTGAGGGCTGCCAGATCATCGAT
R73A	CAGCTGCCTGGTTTACG CAGCTGATACCCAGAC	GTCTGGGTATCAGCTG CGTAAACCAGGCAGCTG
D75A	CTGGTTTACCGAGCTGCTACCCAGACATACCAG	CTGGTATGTCTGGGTAGCAGCTCGGTAAACCAG
P81A	ACCCAGACATACCAGGCTTATAACAAAGACTGG	CCAGTCTTTGTTATAAGCCTGGTATGTCTGGGT
K84A	TACCAGCCTTATAACG CAGACTGGATTAAAGAG	CTCTTTAATCCAGTCTG CGTTATAAGGCTGGTA
W86A	CCTTATAACAAAGACG CGATTAAAGAGAAGATC	GATCTTCTCTTTAATCG CGTCTTTGTTATAAGG
E89A	CAAAGACTGGATTAAAGCGAAGATCTACGTGCTCC	GGAGCACGTAGATCTTCGCTTTAATCCAGTCTTTG

^a^Mutated nucleotides are underlined.

^b^Primers used to add substitutions S24A and S24D to forms T18A and T18D, respectively, due to their overlapping with the latter mutations.

### Maintenance and transfection of HeLa cells

The HeLa cell line was purchased from the European Collection of Cell Cultures (ECACC 93021013) and was cultured in DMEM supplemented with 10% fetal bovine serum, penicillin (100 U/ml) and streptomycin (100 µg/ml) at 37°C in a humidified atmosphere containing 5% CO_2_. For transfection with the FuGENE HD reagent (Roche), 1.5 x 10^5^ cells were seeded on a sterile 16-mm square glass coverslip in a 35-mm plate and the manufacturer’s recommended protocol for transfection with plasmid DNA in the presence of serum was followed. Two days after transfection, cells were washed with phosphate-buffered saline and analyzed by confocal microscopy. For DNA staining, Hoechst 33342 (Sigma) had been added at the concentration of 1 µg/ml for 20 min just before cells were washed and analyzed.

### Confocal microscopy

Images of cells were acquired on a Nikon A1 confocal laser scanning microscope equipped with a Plan Apochromat VC 60x/1.40 Oil DIC objective. For detecting green fluorescence of EGFP-tagged ERHs, 488 nm excitation line and a 525/25 nm band-pass emission filter were used, for detecting red fluorescence of mCherry-tagged proteins (SC35, PDIP46/SKAR and Ciz1), 561 nm excitation line and a 595/50 nm band-pass emission filter were used, and for detecting blue fluorescence of Hoechst 33342, 405 nm excitation line and a 450/50 nm band-pass emission filter were used.

### GST pull-down assay

GST-PDIP46/SKAR[L7] and GST-Ciz1[B] were expressed from pGEX-4T-1/PDIP46/SKAR[L7] and pGEX-4T-2/Ciz1[B], respectively, and wild-type and substituted forms of ERH were expressed from pQE30/ERH-FLAG, pQE30/ERH[H3A Q9A]-FLAG, pQE30/ERH[E37A T51A]-FLAG, and pQE30/ERH[H3A Q9A E37A T51A]-FLAG in *E. coli* XL1 Blue MRF' by induction at OD_600_ of 0.7-0.8 with 1 mM IPTG for 3-4 h at 37°C. GST-PDIP46/SKAR[L7]- or GST-Ciz1[B]-coated beads were obtained using Glutathione-Agarose by batch purification as suggested by the supplier (Sigma). ERH-FLAG, ERH[H3A Q9A]-FLAG, ERH[E37A T51A]-FLAG, and ERH[H3A Q9A E37A T51A]-FLAG were purified using HIS-Select Nickel Affinity Gel (Sigma) according to the manufacturer’s protocol for batch adsorption followed by dialysis against binding buffer (20 mM HEPES, pH 7.4, 50 mM NaCl, 75 mM KCl, 1 mM DTT, 1 mM EDTA, 10% (v/v) glycerol, 0.05% (v/v) Triton X-100). The GST-PDIP46/SKAR[L7]- or GST-Ciz1[B]-coated beads were incubated with equal amounts of ERH-FLAG, ERH[H3A Q9A]-FLAG, ERH[E37A T51A]-FLAG or ERH[H3A Q9A E37A T51A]-FLAG followed by western blotting and enhanced chemiluminescence detection as described previously [10].
